# Exploitation of ammonia-inducible promoters for enzyme overexpression in *Bacillus licheniformis*

**DOI:** 10.1093/jimb/kuab037

**Published:** 2021-06-14

**Authors:** Peili Shen, Dandan Niu, Kugen Permaul, Kangming Tian, Suren Singh, Zhengxiang Wang

**Affiliations:** College of Biotechnology, Tianjin University of Science and Technology, Tianjin 300457, China; Department of Biological Chemical Engineering, College of Chemical Engineering and Materials Science, Tianjin University of Science and Technology, Tianjin 300457, China; Department of Biotechnology and Food Science, Faculty of Applied Sciences, Durban University of Technology, P. O. Box 1334, Durban 4001, South Africa; Department of Biological Chemical Engineering, College of Chemical Engineering and Materials Science, Tianjin University of Science and Technology, Tianjin 300457, China; Department of Biotechnology and Food Science, Faculty of Applied Sciences, Durban University of Technology, P. O. Box 1334, Durban 4001, South Africa; College of Biotechnology, Tianjin University of Science and Technology, Tianjin 300457, China; Department of Biological Chemical Engineering, College of Chemical Engineering and Materials Science, Tianjin University of Science and Technology, Tianjin 300457, China

**Keywords:** *Bacillus licheniformis*, Transcriptome, Ammonia-inducible promoter, Enzyme expression, α-amylase

## Abstract

Ammonium hydroxide is conventionally used as an alkaline reagent and cost-effective nitrogen source in enzyme manufacturing processes. However, few ammonia-inducible enzyme expression systems have been described thus far. In this study, genomic-wide transcriptional changes in *Bacillus licheniformis* CBBD302 cultivated in media supplemented with ammonia were analyzed, resulting in identification of 1443 differently expressed genes, of which 859 genes were upregulated and 584 downregulated. Subsequently, the nucleotide sequences of ammonia-inducible promoters were analyzed and their functionally-mediated expression of *amyL*, encoding an α-amylase, was shown. TRNA_RS39005 (*copA*), TRNA_RS41250 (*sacA*), TRNA_RS23130 (*pdpX*), TRNA_RS42535 (*ald*), TRNA_RS31535 (*plp*), and TRNA_RS23240 (*dfp*) were selected out of the 859 upregulated genes and each showed higher transcription levels (FPKM values) in the presence of ammonia and glucose than that of the control. The promoters, P*_copA_* from *copA*, P*_sacA_* from *sacA*, P*_pdpX_* from *pdpX*, P*_ald_* from *ald*, and P*_plp_* from *plp*, except P*_dfp_* from *dfp*, were able to mediate *amyL* expression and were significantly induced by ammonia. The highest enzyme expression level was mediated by P*_plp_* and represented 23% more α-amylase activity after induction by ammonia in a 5-L fermenter. In conclusion, *B. licheniformis* possesses glucose-independent ammonia-inducible promoters, which can be used to mediate enzyme expression and therefore enhance the enzyme yield in fermentations conventionally fed with ammonia for pH adjustment and nitrogen supply.

## Introduction

Proton motive force (PMF) is the energy source involving the movement of protons across the cell membrane creating an electrochemical potential (Abad, 2011). PMF is an important impetus to promote translocation of enzymes across the membrane (Cranford-Smith & Huber, 2018; Tsirigotaki et al., 2017). During this process, net consumption of protons in the cytoplasmic compartment and net release or accumulation of protons exterior to the cytoplasmic membrane occurs (Srinivasan & Mahadevan, 2010). Therefore, the pH of the enzyme fermentation broth during fermentation should be well-adjusted by an alkaline solution to maintain a balance of Na^+^ and protons (Baskaran & Muthukumarasamy, 2017; Padan et al., 2005), using reagents such as sodium hydroxide or ammonium hydroxide.

Ammonium hydroxide is not merely an ideal alkaline agent, but also serves as a cost-effective nitrogen source frequently used in the enzyme manufacturing processes. When ammonia is dissolved in water, the water molecules donate a proton to the NH_3_ molecule and leads to the formation of an ammonium cation (NH_4_^+^) and a hydroxide ion (OH^−^). Ammonium is also one of the major sources of nitrogen for bacteria, yeasts, and plants (Detsch & Stulke, 2003; Liu & von Wirén, 2017; Magasanik & Kaiser, 2002). The fate of ammonium hydroxide during the enzyme fermentation is: (1) its OH^−^ controls the amount of H^+^ that accumulates when the enzyme molecules are translocated outside of the cytoplasmic membrane, (2) its NH_4_^+^ is taken up and incorporated to yield glutamine and/or glutamate (Detsch & Stulke, 2003). This ensures the preferential utilization of glutamine as the nitrogen source that can be used with the lowest energy cost (Detsch & Stulke, 2003).

The molecular mechanism and metabolic pathway for bacteria utilizing ammonium as a nitrogen source have been well-demonstrated (Atkinson & Ninfa, 2010). In *Bacillus subtilis*, the assimilation of ammonium depends on the ATP-fueled glutamine synthetase–glutamate synthetase cycle, and its transmembrane transport depends on the homotrimeric ammonium transporter AmtB (Gunka & Commichau, 2012). The *nrgA* gene encoding AmtB together with *nrgB*, encoding a p-II like protein GlnK, forms the *nrgAB* operon, which is conserved in many organisms and controls the ammonium uptake (Thomas et al., 2001).

*Bacillus licheniformis* is an industrially important host used for overexpression and preparation of many enzymes at commercial scale (Niu & Wang, 2007). Ammonium hydroxide is conventionally used as a pH-controlling agent. Previous literature investigated the response of *B. licheniformis* to medium pH upshifts (Hornbaek et al., 2004), heat and ethanol stress (Voigt et al., 2013), osmotic challenges (Schroeter et al., 2013), acetoin stress (Yuan et al., 2019a), peroxide stress (Schroeter et al., 2011), oligosaccharides elicitors (Reffatti et al., 2014), and glucose, nitrogen, and phosphate starvation (Hoi le et al., 2006; Voigt et al., 2007). All the above parameters had been well-studied through transcriptomics, metabolomics and proteomics. Up to now, there seems to be a lack of information on the response of *B. licheniformis* to ammonia.

The aim of this study was to exploit an ammonia-inducible promoter for enzyme expression in *B. licheniformis*. The response of *B. licheniformis* to ammonia was transcriptomically analyzed, putative ammonia-inducible promoters were functionally identified, and their capacity for mediating enzyme expression was analyzed; and scale-up fermentation was performed in 5 l bioreactor. It was found that *B. licheniformis* has glucose-independent ammonia-inducible promoters, which can be used to mediate enzyme expression and therefore enhance the enzyme yield by ammonia conventionally fed for acidic pH adjusting and nitrogen supply.

## Materials and Methods

### Strains, Plasmids, and Cultivation

The bacterial strains and plasmids used in this study are listed in Table 1. *Escherichia coli* JM109 was used as the host for gene manipulation. *B. licheniformis* BL-109 was derived from *B. licheniformis* CBBD302 (Niu et al., 2009) by deletion of the thermophilic α-amylase encoding gene *amyL* and used as the host cell for gene expression. pHY-amyL was constructed in previous studies (Niu & Wang, 2007). Both *E*. *coli* JM109 and *B. licheniformis* BL-109 were cultivated at 37°C in LB medium (1% tryptone, 1% NaCl, 0.5% yeast extract) supplemented with 20 μg/ml kanamycin when necessary.

**Table 1.** The Strains and Plasmids Used in This Study

Strain/Plasmid	Description	Source
Strain		
*Bacillus licheniformis* CBBD302	*B. licheniformis* CBB0302, deleted type I RMS locus	(Niu et al., 2009)
*B. licheniformis* BL-109	*B. licheniformis* CBBD302, ∆*amyL*	Lab stock
*Escherichia coli* JM109	*endA1*, *recA1*, *gyrA96*, *thi*, *hsdR17*, *rel*A1, *sup*E44, λ−, Δ(*lac-pro*AB), [Fʹ, *tra*D36, *pro*AB, *laqI^q^*ZΔM15]	(Yanisch-Perron et al., 1985)
*B. licheniformis* RBA-0	*B. licheniformis* BL-109, harboring pHY000-amyL	This study
*B. licheniformis* RBA-1	*B. licheniformis* BL-109, harboring pHY001-amyL	This study
*B. licheniformis* RBA-2	*B. licheniformis* BL-109, harboring pHY002-amyL	This study
*B. licheniformis* RBA-3	*B. licheniformis* BL-109, harboring pHY003-amyL	This study
*B. licheniformis* RBA-4	*B. licheniformis* BL-109, harboring pHY004-amyL	This study
*B. licheniformis* RBA-5	*B. licheniformis* BL-109, harboring pHY005-amyL	This study
*B. licheniformis* RBA-6	*B. licheniformis* BL-109, harboring pHY006-amyL	This study
Plasmid		
pHY-amyL	Harboring P*_amyL_*/S*_amyL_* from *B. licheniformis* and thermostable α-amylase gene, Km^R^	(Niu & Wang, 2007)
pHY000-amyL	Deleted P*_amyL_* of pHY-amyL, Km^R^	This study
pHY001-amyL	Replaced P*_amyL_* of pHY-amyL by P*_copA_*, Km^R^	This study
pHY002-amyL	Replaced P*_amyL_* of pHY-amyL by P*_sacA_*, Km^R^	This study
pHY003-amyL	Replaced P*_amyL_* of pHY-amyL by P*_ald_*, Km^R^	This study
pHY004-amyL	Replaced P*_amyL_* of pHY-amyL by P*_pdbX_*, Km^R^	This study
pHY005-amyL	Replaced P*_amyL_* of pHY-amyL by P*_plp_*, Km^R^	This study
pHY006-amyL	Replaced P*_amyL_* of pHY-amyL by P*_dfp_*, Km^R^	This study

### Sample Preparation, Sequencing, and Data Analysis

Before mRNA isolation, the cells of *B. licheniformis* CBBD302 were pretreated as following steps. *B. licheniformis* CBBD302 were propagated in 50 ml LB medium in 250-ml shake flasks at 37°C with agitation of 200 rpm for 15 hr until the culture reached mid-log phase. The cells were collected by centrifugation at 5,000 × *g* for 5 min and resuspended in 5 ml fresh LB medium; and 500 μL was then transferred into 50 ml LBGN (LB medium supplemented with 1% glucose and 0.1% ammonium hydroxide). The mixture was incubated at 37°C and 200 rpm for 5 hr. The cells were collected by centrifugation at 5,000 × *g* for 5 min and immediately frozen in liquid nitrogen and stored at −70°C for RNA isolation. In parallel, *B. licheniformis* CBBD302 cells were prepared as a control grown under the same conditions, but in LBG (LB, 1% glucose).

Total mRNA was prepared by Gene Denovo Biotechnology Co., Ltd (Guangzhou, China) and subsequently sequenced on the Illumina sequencing platform. All obtained clean reads were mapped to the *B. licheniformis* ATCC 14580 genome (NCBI database; NC_006270.3) by Tophat 2 (Kim et al., 2013). The gene expression was normalized by using FPKM (fragments per kilobase of transcript per million mapped reads) (Trapnell et al., 2010). The software edgeR was used for the analysis of differentially expressed genes (DEGs) between control and test samples using the following parameters: false discovery rate (FDR) <0.05 and absolute value of log2 fold change >1 (Robinson et al., 2010).

Kyoto Encyclopedia of Genes and Genomes (KEGG) enrichment was conducted to investigate the main metabolic pathways related to the DEGs (Minoru et al., 2008). All DEGs were subjected to the KEGG database (http://www.genome.jp/kegg) to identify biological functionality.

The putative binding sites for σ-factors and transcription factors (TFs) were analyzed using DBTBS (http://dbtbs.hgc.jp/) (Makita et al., 2004). The sequence of the open reading frames immediately upstream was predicted using Softberry Inc. (BPROM) to identify promoters (http://linux1.softberry.com/berry.phtml?topic=bprom&group=programs&subgroup=gfindb).

### Genetic Manipulation

Chromosomal DNA isolation, PCR amplification, transformation, plasmid extraction, restriction endonuclease digestion, ligation, and nucleotide sequencing and analysis were carried out according to established protocols (Sambrook & Russel, 2001). Primers (Table 2) were designed and chemically synthesized by Sangon Biotech (Shanghai) Co., Ltd. based on the sequence of ∼1.0 kb upstream regions from the first nucleotide of the SD sequence of the selected DEGs.

**Table 2.** Primers Used in This Study

Primer	Nucleotide sequence*^a^* (5ʹ→3ʹ)^*^	Target sequence
CopA-F	GCCGAAGATAATGACGCTGA	P*_copA_*
CopA-R	TTGTCTAGACAAATGAAATATGATTCTACCG	
SacA-F	GTTCCATTATCTCCGTCAGCAT	P*_sacA_*
SacA-R	GAGTCTAGACACTTGATTTAAGAGTCTTATG	
Ald-F	ATGACTTCCTGGATTGGGATACAT	P*_ald_*
Ald-R	AGCTCTAGAGTGGTTTGATGATATTGATCG	
PdbX-F	CGATACCATTCCAAGAAACAAGG	P*_pdbX_*
PdbX-R	AAATCTAGATTTTTTTTAAAAAAAAGCAGAAAAG	
Plp-F	AAGACATTCCAGTCGTAACCTC	P*_plp_*
Plp-R	CGCTCTAGAGCGTTTTCTTTATTTTGGTC	
Dfp-F	AACTTCTTCGTGAGCCGTTCAAC	P*_dfp_*
Dfp-R	GAATCTAGATTCGTTTTTTGTATCTGCCTTA	
PHY-UpR	GCCCATTCTTTAAACGGAAATTC	Removal of the promoter sequence of pHY-*amyL*
PHY-DnPA	CATCCTAGGATGTTTGCAAAACGATTCAAAAC	

^*^Underlined base-pairs TCTAGA/CCTAGG represent the *Xba*I/*Avr*II restriction sites, respectively.

The selected promoter candidates were amplified with primers listed in Table 2 by PCR using the chromosomal DNA of *B. licheniformis* CBBD302 as template. The amplified products were purified and digested with *Xba*I. It was then ligated with the *Avr*II-digested reverse PCR product of pHY-amyL using primers pHY-UpR and pHY-DnPA. The ligation mixtures were transformed into *E. coli* JM109 and the resultant recombinant plasmids (Table 1) were transferred into *B. licheniformis* BL-109 using the method described by Xu et al. (2004) and the transformants were confirmed by plasmid DNA extraction and restriction digestion analysis. Additionally, the reverse PCR product of pHY-amyL was cyclized and transformed into *E. coli* JM109 to form a new plasmid (pHY000-amyL) without a promoter sequence. Plasmid pHY000-amyL was transformed into *B. licheniformis* BL-109 yielding a recombinant strain used as the negative control.

### Fermentation Experiments

Recombinant *B. licheniformis* strains were cultivated in 50 ml LB medium supplemented with 20 μg/ml kanamycin in 250-ml flasks at 37°C and 200 rpm for 15–18 hr until late exponential phase. A 10% inoculum was then added into 50 ml fermentation media and incubated at 37°C and 200 rpm for up to 120 hr. The carbon source in the fermentation medium was modified from previous studies by changing lactose to glucose (Niu et al., 2009). During the fermentations, 0.0125% (wt/vol) of ammonium hydroxide was added every 24 hr. The supernatants were harvested by centrifugation and then used for enzyme assays.

Fermentations conducted in a 5-L fermenter with 2.5 l starting volumes were carried out as described previously (Niu et al., 2009). The pH of the fermentation broths was automatically controlled at 6.05 ± 0.05 by feeding 25% sodium hydroxide or 25% ammonium hydroxide. The fermentation feeding 25% ammonium hydroxide was set as control. The enzyme activities and cell mass were measured every 8 hr in triplicate.

### Analytical Procedures

The activity of α-amylase was assayed by the DNS method, as described previously (Liu et al., 2010), with modifications. In brief, the reaction mixture consisted of 500 μL starch solution (1.0 g/L) in 250 μL 100 mmol/L citric acid/Na_2_HPO_4_ buffer (pH 6.0) and 500 μL crude enzyme. After incubation at 70°C for 30 min, 100 μL of iodine reagent (5 mmol/L I_2_, 5 mmol/L KI, 0.2 mol/L HCl) was added. The absorbance at 550 nm (A_550_) was measured using a SP-2012 UV spectrophotometer (Shanghai Spectrum Instruments, China). One unit of α-amylase activity was defined as the amount of enzyme required to liberate one µmol glucose per minute. The optical density (600 nm) of the cultures were also measured with an SP-2012 UV spectrophotometer. The mass of the fermentation broth pellets was used to estimate the cell mass. These assays were conducted in triplicate.

## Results and Discussion

### Transcriptomic Analysis Revealed the Landscape of *B. licheniformis* Response to Ammonia

To gain a global view of the transcriptional response to ammonia in *B. licheniformis* CBBD302, the transcriptome profiles of *B. licheniformis* CBBD302 grown in LB medium, supplemented with 1% glucose and 0.1% ammonia or 1% glucose only, were compared. As a consequence, high quality RNA-seq data was generated, comprising 16,471,120 (supplemented with 1% glucose and 0.1% ammonia) and 15,436,656 (only 1% glucose) high-quality clean reads (total reads) obtained after filtering raw reads and removing rRNA mapped reads. Of these, 97.07% and 97.13% of all reads were uniquely mapped to the reference genome of *B. licheniformis* ATCC 14580 and approximately 80.87% and 79.02% of the expressed genes had a sequencing coverage of 80–100%.

**Fig. 1.**
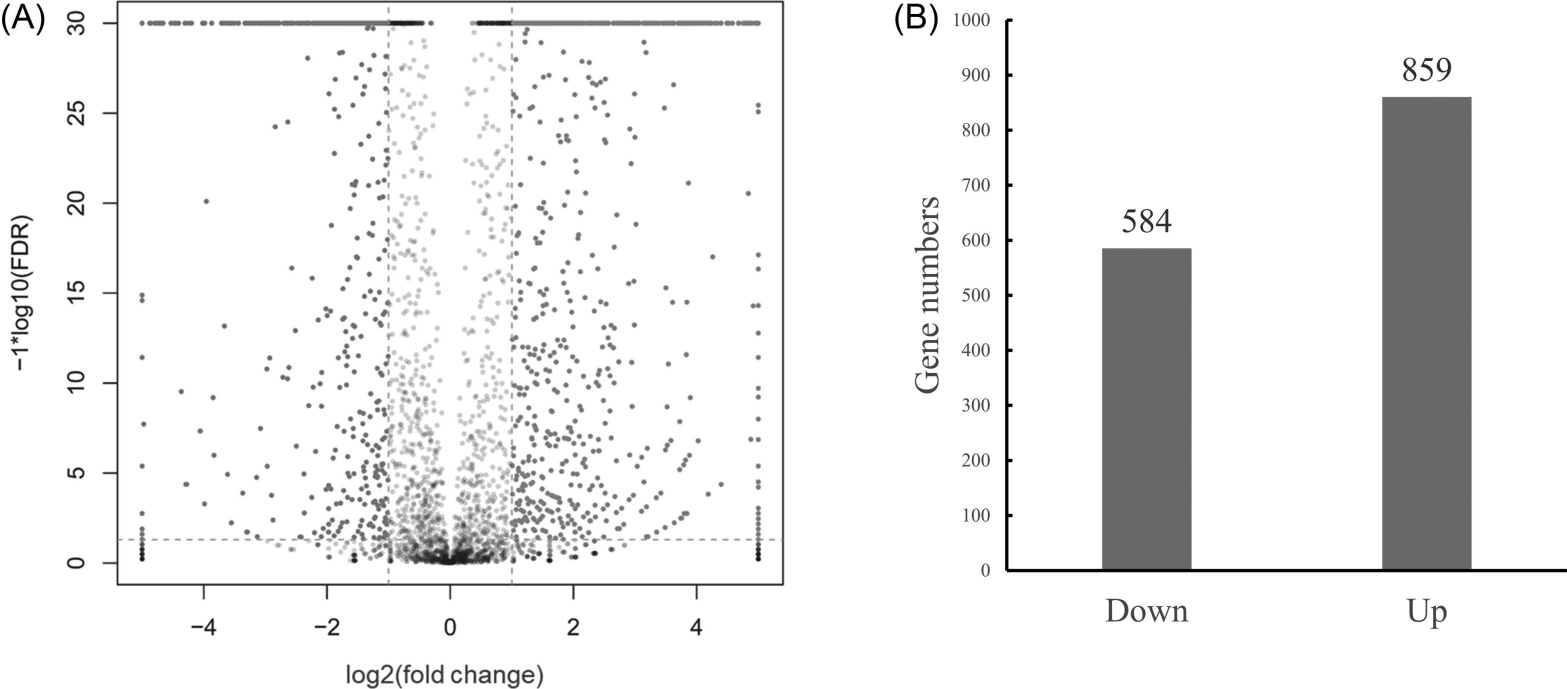
The differentially expressed genes in *B. licheniformis* CBBD302 in response to ammonia. (A) The overall scatter of gene transcription by volcano plots. Middle and bottom (black dots, absolute value of log2 fold change˂1): non-significantly regulated genes; Right (black dots, log2 fold change >1, FDR < 0.05: significantly upregulated genes; Left (gray dots, log2 fold change ˂−1), FDR < 0.05: significantly downregulated genes. (B) The number of genes with up- or downregulation. Up: the number of genes upregulated; Down: the number of genes downregulated.

The DEGs were extracted from the RNA-seq data and 1,443 DEGs were identified at a threshold of absolute value of log2 fold change >1 and an FDR <0.05. Eight hundred fifty nine of 1,443 genes were significantly upregulated and 584 genes were significantly downregulated (Fig. 1; Supplementary Table S1). When absolute value of log2 fold change >1, FDR <0.01, 815 genes were upregulated while 554 genes were downregulated (Supplementary Table S1).

**Fig. 2.**
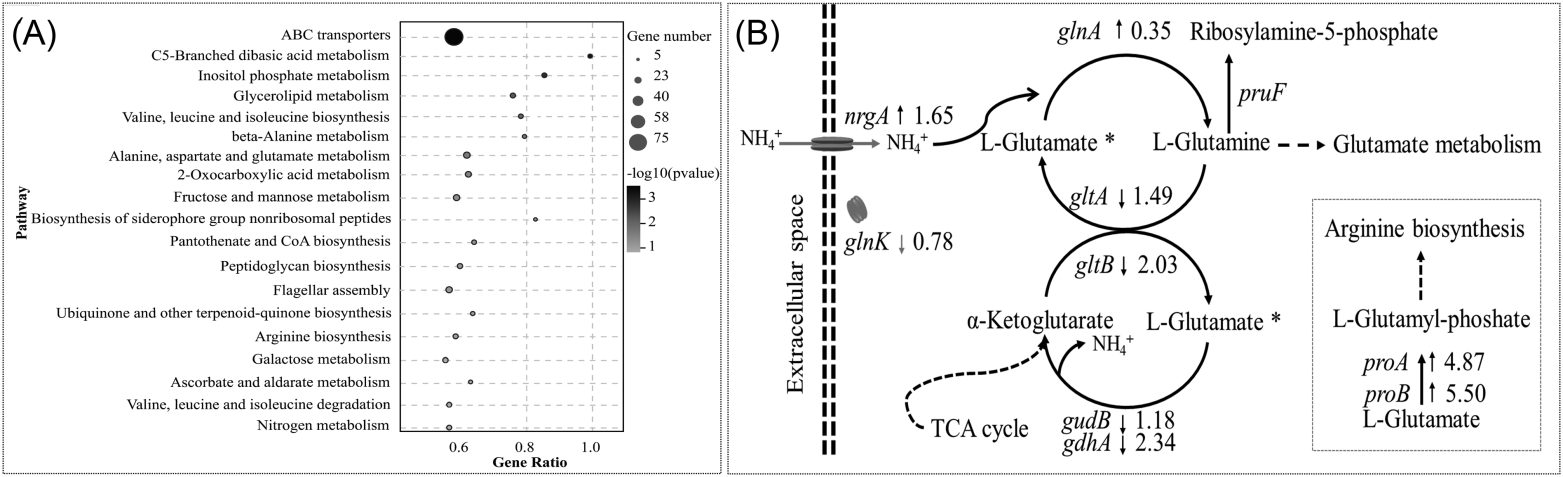
KEGG classification of the DEGs in the *B. licheniformis* CBBD302 response to ammonia. (A) The top 20 pathways enriched in KEGG classification after incubation with ammonia; (B) The changes of transcription level of ammonium metabolism related coding genes, short up-arrow: indicates that the marked gene was upregulated while the short down arrow means the marked gene was downregulated; gene *pruF* was not changed; ^*^The embedded box illustrates the downstream pathway of L-glutamate.

The DEGs were further analyzed by KEGG and the results are summarized in Fig. 2. Five hundred forty four of 1443 DEGs were successfully matched to 104 of 115 different KEGG pathways (Supplementary Table S2). The DEGs were overrepresented in the following pathways: “ABC transporters,” “C5-branched dibasic acid metabolism,” “inositol phosphate metabolism,” “phosphotransferase system (PTS),” “valine, leucine, and isoleucine biosynthesis,” “beta-alanine metabolism,” “alanine, aspartate, and glutamate metabolism,” and “2-oxocarboxylic acid metabolism” (Fig. 2A). Furthermore, the transcription levels of 12 genes involved in nitrogen metabolism and five others main key ammonium metabolism-related genes were significantly changed (Table 3). The intracellular nitrogen metabolic process had been altered for using ammonia as the nitrogen source. Notably, the transcription level of the ammonium transporter coding gene *nrgA* was upregulated while *nrgB* showed little change. On the other hand, the gene *glnA* encoding glutamine synthetase catalyzing the condensation of glutamate and ammonium to form glutamine (Gunka & Commichau, 2012) was slightly upregulated to facilitate the assimilation of ammonium (Fig. 2B). The metabolism of *B. licheniformis* CBBD302 was therefore significantly changed after it was cultivated with ammonium hydroxide.

**Table 3.** The Transcription Regulation of Nitrogen Related Genes

Gene ID	CK-FPKM	T1-FPKM	Log2 fold change	Symbol	Description
**Nitrogen metabolism**					
TRNA_RS23310	4.84	27.73	2.52	*nasA*	Nitrate transporter
TRNA_RS23885	1.35	3.59	1.41	*nasB*	Nitrite reductase large subunit
TRNA_RS23890	0.4	1.62	2.02	*nasC*	Nitrite reductase
TRNA_RS23895	87.28	26.61	−1.71	*nasD*	Assimilatory nitrite reductase (subunit)
TRNA_RS31810	7.3	2.38	−1.62	*norB*	Nitric-oxide reductase large subunit
TRNA_RS32205	1,993.08	402.1	−2.31	*gltB*	Glutamate synthase (small subunit)
TRNA_RS32210	1,961.32	696.69	−1.49	*gltA*	Glutamate synthase large subunit GltA
TRNA_RS33580	888.78	393.38	−1.18	*gudB*	Glutamate dehydrogenase
TRNA_RS36165	130.56	25.7	−2.34	*gdhA*	Glutamate dehydrogenase
TRNA_RS37375	71.43	33.17	−1.11	*Csh*	Carbonic anhydrase
TRNA_RS37790	54.5	205.9	1.92	*yrpB*	Nitronate monooxygenase
TRNA_RS39775	33.43	160.84	2.27	*–*	Carbonic anhydrase, prokaryotic YvdA
**Ammonium metabolism**					
TRNA_RS40610	4.68	14.73	1.65	*nrgA*	Ammonium transporter
TRNA_RS40615	129.22	84.29	−0.62	*nrgB*	Nitrogen-regulated PII-like protein
TRNA_RS31370	1079.57	1,376.9	0.35	*glnA*	Glutamine synthetase
TRNA_RS22870	11.76	6.85	−0.78	*glnK*	Two-component sensor histidine kinase GlnK
TRNA_RS28910	2.06	1.58	−0.38	*tnrA*	Transcriptional regulator

However, the genes related to nitrogen or ammonium metabolism did not show relatively higher FPKM values when *B. licheniformis* CBBD302 was grown in LBGN medium compared to growth in LBG medium. These results indicated that the scope of the search should have been extended to identify ammonia-inducible promoters. Therefore, a global screen of the DEGs was carried out to search for proper candidate genes. As a result, six upregulated genes (3 individual genes and 3 operons) with high log2 fold change (≥3.5) and high FPKM values (≥2,000), when incubated with ammonia while showing low FPKM values (≤350) in the control, were identified from the 859 upregulated genes (Table 4). Only two of the six selected DEGs, TRNA_RS42535 and TRNA_RS41250, were found to match with two different KEGG terms of “taurine and hypotaurine metabolism” and “galactose metabolism,” respectively, based on the result of KEGG cluster analysis.

**Table 4.** The Identified Candidate Promoter Genes

Gene ID	Gene or operon	Gene in operon	CK-FPKM	T1-FPKM	Log2 fold change	Symbol
TRNA_RS39005	Operon	TRNA_RS39005, TRNA_RS39010	233.72	6,623.44	4.82	*copA*
		TRNA_RS41245, TRNA_RS41250, TRNA_RS41255				
TRNA_RS41250	Operon	TRNA_RS31535, TRNA_RS31540	304.15	5,168.93	4.08	*sacA*
TRNA_RS42535	Operon	TRNA_RS23130	37.49	2,115.45	5.82	*ald*
TRNA_RS23130	Gene	TRNA_RS42535	246.39	4,651.72	4.3	*pdbX*
TRNA_RS31535	Gene	TRNA_RS23240	137.77	4,263.28	4.91	*plp*
TRNA_RS23240	Gene	TRNA_RS31535, TRNA_RS31540	172.8	2,005.56	3.54	*dfp*

### Promoter Structure and Sequence Analysis of the Putative Ammonia-Inducible Genes in *B. licheniformis* CBBD302

It is well recognized that if gene transcription occurs at a higher level under favorable, adverse, or threatening conditions, it often contains a promoter with the specific nucleotide sequence that is reactive to the corresponding factors (Song et al., 2016). To further analyze the structure of promoter candidates, approximately 1,000 bp upstream sequences of the target genes were selected to predict the −35, −10 elements and spaces between these two elements with Softberry software and the σ factors; and TFs of the candidates were predicted using the DBTBS database. The results are summarized in Table 5. Three promoters of the candidates (named P*_copA_*, P*_sacA_*, and P*_pbdX_*) were not associated with specific σ factors, two (P*_ald_* and P*_plp_*) were controlled by SigW and P*_dfp_* was controlled by two σ factors, SigW and SigX. CodY is a global transcriptional regulator and it was reported to control more than 100 genes, and could activate them at specific periods (Sonenshein, 2005). Transcription factor araR/araB is involved in arabinose metabolism (Büttcher et al., 1997) and CcpA regulates a large number of genes involved in carbon metabolism, amino acid anabolism, overflow metabolism, and nitrogen assimilation (Wünsche et al., 2012). Additionally, P*_plp_* was the only one that was predicted to have the binding sites for SigW and two putative TFs, AraR and CodY, simultaneously. The coordination of SigW and TFs would enhance the transcription of target genes when needed.

**Table 5.** The Core Elements of Ammonia–Inducible Promoter Candidates

Promoter	Gene ID	Promoter core elements^*^	Sigma factor^**^	TF^**^
P*_copA_*	TRNA_RS39005	TTTC**TTGAAA**TACCCTACAGGGGTA**TGGTAATAT**AAAACCG**A**	N	PurR
P*_sacA_*	TRNA_RS41250	GTAG**TTGACG**AAAGCGTTATCACA**TAATAAAAT**GAAAGCGT**A**	N	CcpA, CodY
P*_ald_*	TRNA_RS23130	ACAT**TTTAAG**CCTTATACCTATC**TTTTGGAAT**TCGTGA**A**	SigW	N
P*_pdbX_*	TRNA_RS42535	TCGC**TTACAT**TTGTTTTTTTAACAA**AGCTATTTT**TTAAA**G**	N	AbrB, CcpA, Xre
P*_plp_*	TRNA_RS31535	GTCT**TTTAAG**TTGATGATTTCACAA**TGATAAAAT**TTTTTTCT**A**	SigW	AraR, CodY
P*_dfp_*	TRNA_RS23240	ACGC**TTGAAA**CAATTTCCTTGCTTTTCC**GTATATAAG**GCAGAT**A**	SigW, SigX	N

^**^The predicted −35, −10 elements are indicated in bold letters with gray background; The single bold letter was predicted as the start site of transcription.

^**^N: no prediction result.

### Ammonia-Inducible Promoters in *B. licheniformis* Functionally Mediated the Expression of α-amylase

**Fig. 3.**
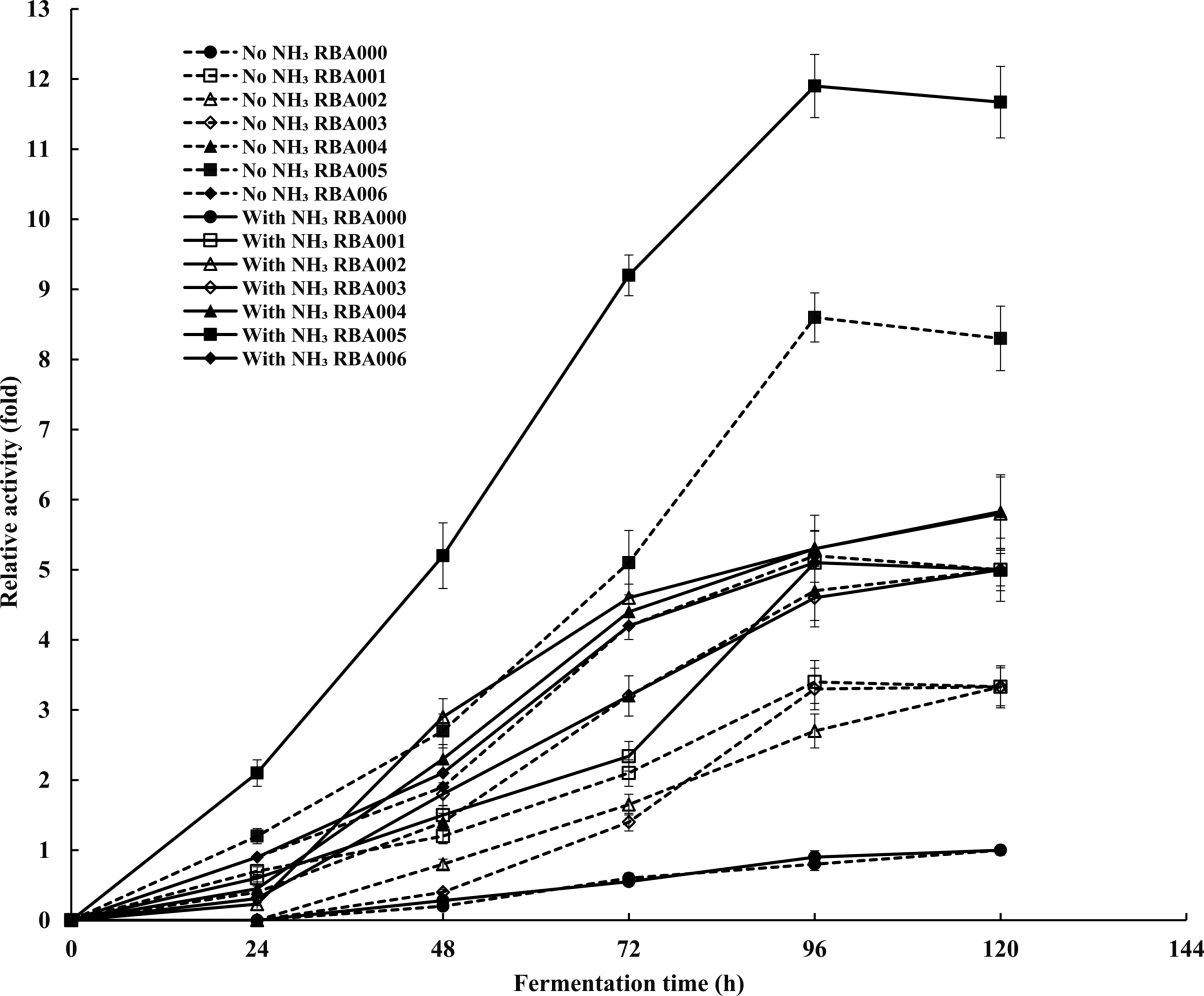
The expression levels of α-amylase mediated by the putative ammonia-inducible promoters. The fermentation was carried out in 250-ml shake flasks with working volumes of 50 ml. The α-amylase activities in fermentation medium with no ammonia (dot line) or added ammonia (solid line) were measured. The enzyme activity of the control, recombinant *B. licheniformis* RBA000, was designated as a relative activity of one. Error bar indicates standard deviation from three parallel experiments.

To test if the selected promoters could mediate gene expression induced by ammonia, a series of recombinant amylase expression plasmids—pHY001-amyL, pHY002-amyL, pHY003-amyL, pHY004-amyL, pHY005-amyL, and pHY006-amyL—were generated by replacing the original promoter P*_amyL_* with the selected promoter candidates. They were then transformed into *B. licheniformis* BL-109, resulting in recombinant transformants: *B. licheniformis* RBA-1, RBA-2, RBA-3, RBA-4, RBA-5, and RBA-6. Based on the highest enzyme activities, all six promoters were able to initiate the transcription of *amyL* and five of them—P*_copA_*, P*_sacA_*, P*_ald_*, P*_pdbX_*, and P*_plp_*—mediated *amyL* expression in an ammonia-inducible manner (Fig. 3), while P*_dfp_* was not significantly induced by ammonia under the experimental condition (Fig. 3). P*_sacA_* mediated *amyL* expression with the maximum induction by ammonia (75%). P*_plp_* had the highest expression level among the six candidates and the enzyme activity was increased by 38% after induction by ammonia. The results showed that five glucose-independent, ammonia-inducible promoters were found in *B. licheniformis*.

### An Integrated Fermentation Process for Enzyme Preparation Was Developed Using Ammonia-Inducible Promoters to Mediate Enzyme Expression

**Fig. 4.**
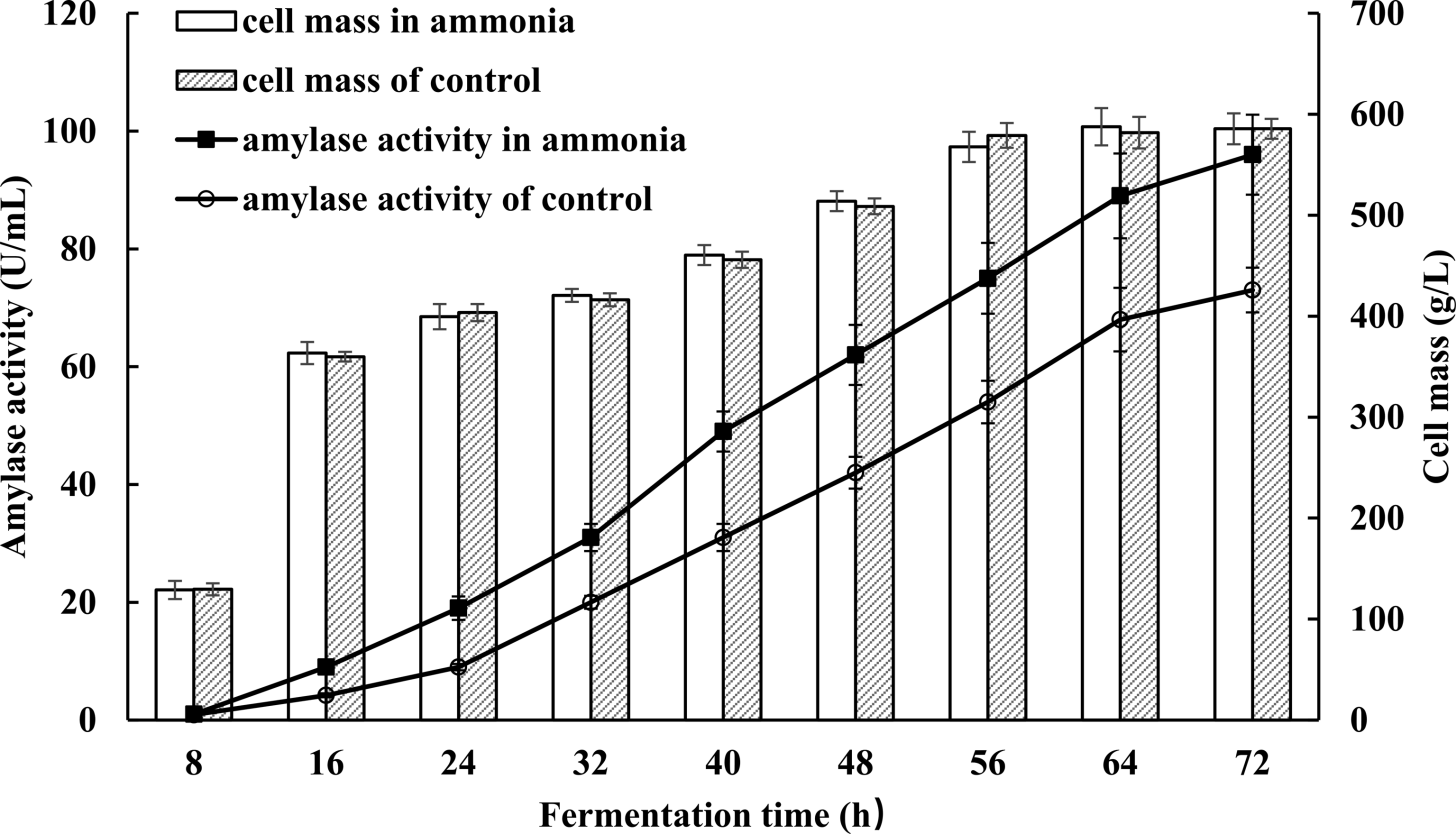
A scaled-up fermentation process using ammonia-inducible promoter P*_plp_*. Error bar indicates standard deviation from three parallel experiments.

To further confirm the improvement fermentation process by the addition of ammonia, two scaled-up fed-batch fermentations were conducted in a 5-L fermenter. *B. licheniformis* RBA-5 harboring the best ammonia-inducible promoter P*_plp_* was selected for large-scale fermentations. The activity of α-amylase in the media fed with ammonium hydroxide was approximately 23% higher than that with sodium hydroxide (Fig. 4). The growth of the cultures, measured by cell mass, was nearly the same in both fermentations.

**Fig. 5.**
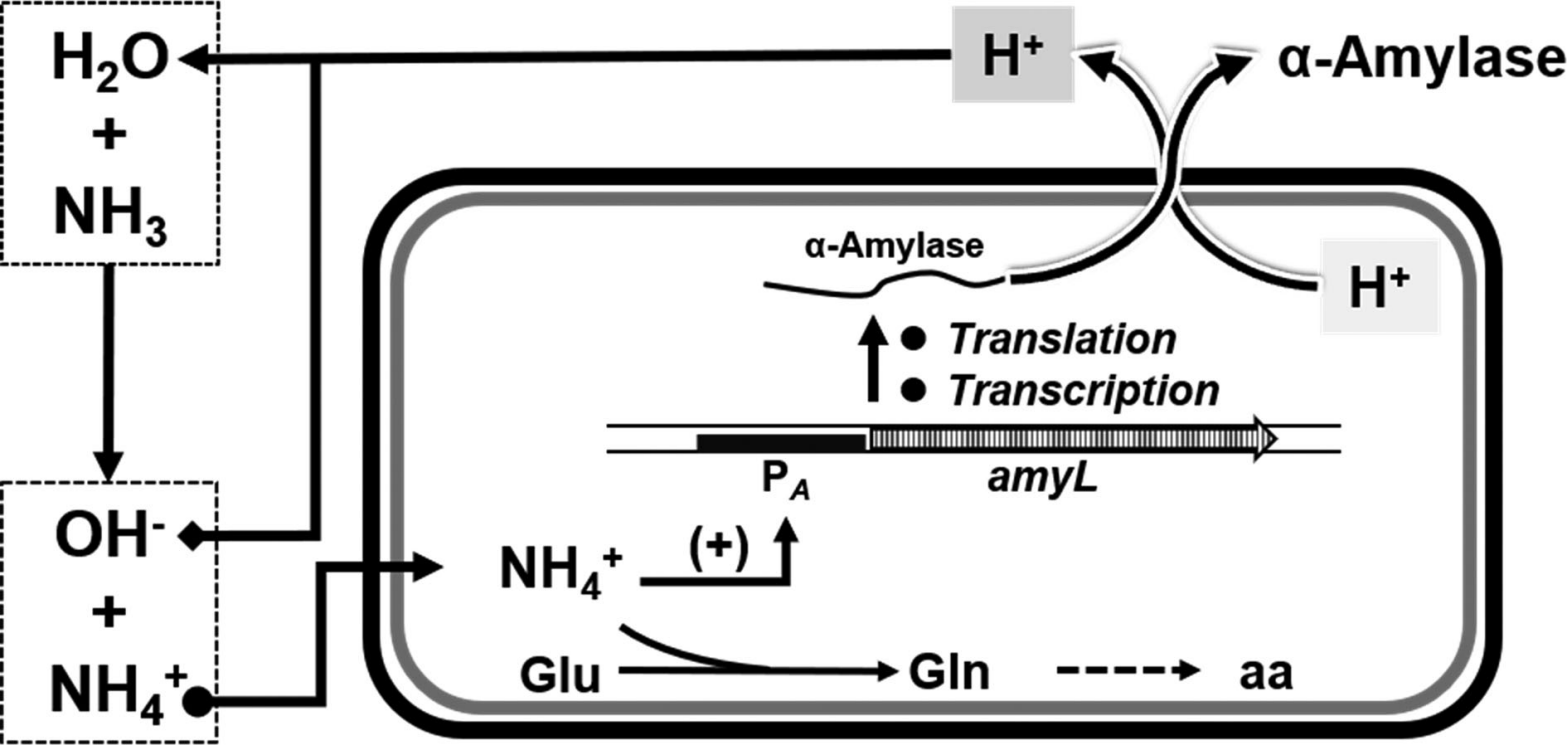
Summary of the roles of ammonia in fermentation process. Firstly, as a pH adjuster to neutralize the protons that accompany the secretion of the enzyme/protein; secondly, as a nitrogen source, ammonium is transferred to Glu (glutamate) formed Gln (glutamine) to supply amidogen to the other amino acids (aa) by deamination; thirdly, as an inducer, ammonia used to trigger the transcription of the target genes in the presence of an ammonia-inducible promoter (P*_A_*).

This result indicates that: (1) ammonia could be used as a preferred nitrogen source for enzyme/protein production, a kind of neutralizer to adjust the pH of the fermentation broth; and (2) more importantly, that ammonia can act as an inducer to elevate the gene transcription levels, thus improving the expression levels of the target enzyme (Fig. 5).

The results of this study provide clear evidence that an ammonia-inducible expression system has been developed. Despite this increase in expression using ammonia-inducible promoters, further enhancement of expression is still possible. Gene regulation is a complicated process and many factors can result in low expression levels. For instance, special sequences like palindrome sequences found between promoter and genes sequences (Yuan et al., 2019b), the change of culture conditions (Liao et al., 2015), different σ factors and TFs also affect the gene expression (Liu et al., 2017). In future, studies involving optimizing the sequence of the -35 and -10 regions to conservative ones (Phan et al., 2012), modifying the RBS sequence (Stammen et al., 2010) and/or synergy with a stronger promoter to form a double-promoter system (Öztürk et al., 2017), could be employed to improve the expression by the ammonia-inducible promoters identified in this study.

## Conclusion

The transcriptomic profile of *B. licheniformis* grown in ammonia was characterized and five glucose-independent ammonia-inducible promoters were functionally identified. By using these ammonia-inducible promoters and ammonia as inducer, the enzyme expression level was significantly improved compared to the control. To the best of our knowledge, this is the first report that ammonia can be employed to mediate enzyme overexpression based on the ammonia-inducible promoters, and therefore enhance the enzyme yield using ammonia conventionally-fed for adjusting acidic pH and as a nitrogen supply.

## Supplementary Material

kuab037_Supplemental_File

## Data Availability

All data generated or analyzed during this study are available within the article and its supplementary material.
